# Influence of training status on cardiac and vascular functioning in young recreational and competitive male rowers

**DOI:** 10.3389/fped.2023.1162594

**Published:** 2023-04-06

**Authors:** Lavinia Falcioni, Maria Chiara Gallotta, Carlo Baldari, Ludovica Cardinali, Matteo Campanella, Dafne Ferrari, Laura Guidetti, Marco Meucci

**Affiliations:** ^1^ Department of Movement, Human and Health Sciences, University of Rome “Foro Italico”, Rome, Italy; ^2^ Department of Physiology and Pharmacology “Vittorio Erspamer”, Sapienza University of Rome, Rome, Italy; ^3^ Department of Theoretical and Applied Sciences, eCampus University, Novedrate, Italy; ^4^ Departement of Unicusano, Università Degli Studi Niccolò Cusano, Rome, Italy; ^5^ Department of Health and Exercise Science, Appalachian State University, Boone, NC, United States

**Keywords:** cardiovascular health, non-invasive measurements, rowing training, young athletes, rower athletes

## Abstract

**Introduction:**

The aim of the study was to investigate the influence of training status on cardiovascular function in young male recreational and competitive rowers.

**Methods:**

Ejection duration in percentage to the heart rate period (ED%), subendocardial viability ratio (SEVR), augmentation index at 75 bpm (AIx75) and carotid to femoral pulse wave velocity (cf-PWV) of competitive rowers (CR) (age 17.6 ± 4.1 years), recreational rowers (RR) (age 16.7 ± 2.70 years) and athletes practicing other recreational sports (ORS) (age 15.3 ± 1.4 years) were assessed.

**Results:**

ED% was lower in CR compared to ORS (31.9 ± 3.9% vs. 38.4 ± 4.8%; *p* = 0.026) and cf-PWV was higher in CR compared to ORS (5.5 ± 1.0 m/s vs. 4.7 ± 0.5 m/s; *p* = 0.032). SEVR was higher in CR compared to RR and ORS (165.8 ± 33.7% vs. 127.4 ± 30.4% and 128.3 ± 27.8%; *p *= 0.022) and AIx75 was lower in CR compared to RR and ORS (−15.7 ± 8.6% vs. 1.2 ± 9.9% and 1.5 ± 9.1; *p* = 0.001).

**Discussion:**

Healthy, young competitive male rowers reported higher myocardial performance and better cardiovascular health than recreational athletes. Interpretations of cf-PWV in competitive rowers should be performed alongside other cardiovascular indicators.

## Introduction

Rowing is considered a unique sport due to the combination of high intensity aerobic and muscular effort that is required during a race. The continuous exertion force using large muscle groups leads rowers to gain both large aerobic power and muscle strength (1, 2). Research has shown that the cardiovascular system of this type of athletes may undergo significant remodelling changing both the structure and function of heart and arteries (3–8).

Previous studies conducted in adults have shown that professional rowing may induce an increase in cardiac volume and mass and that these adaptations differ between male and female athletes (3, 9–11). Although cardiac remodelling occurs in both competitive and recreational rowers, this sport seems to increase specific aspects of cardiac structure and function in elite adult athletes consequently to the higher volume and intensity of training (12). These morphological adaptations can improve the mechanical performance of the heart which consequently increases the systolic flow velocity, the emptying velocity of the left ventricle, and the maximum volume ejected during the left ventricle systole (5, 13). Additionally, recreational and competitive adult rowers reported favourable elastic properties of the arteries and larger vascular diameters (5–8, 14). However, there is also inconclusive evidence on the impact of rowing on cardiovascular functions. Other studies have shown that this discipline may exhibit no-effects or negative effects on vascular characteristics in competitive and recreational adults (8, 15–17). Moreover, the fact that athletes undergo high-intensity training from an early age to compete professionally (18, 19) suggests that more research in adolescent rowers is needed to uncover the cardiovascular adaptations occurring at an early age. Research in adolescents reported that, among different disciplines, competitive male rowers show the highest left ventricular wall thickness and cavity dimension (4). However, little is known about the vascular adaptations in young rowers as the majority of research has been conducted on adults (7, 15). Thus, the aim of this study was to investigate the influence of training status on cardiovascular functioning in young recreational and competitive male rowers.

## Materials and methods

### Participants

Twenty-eight, young, healthy males (age = 16.3 ± 2.7 years) were recruited for this study. Participants were divided in three groups: competitive rowers (CR, *N* = 8), recreational rowers (RR, *N* = 8) and other recreational sports (ORS, *N* = 12). The CR group was composed of athletes with 6.0 ± 3.9 years of training experience, who trained at least 5 times per week (12.8 ± 2.3 h per week, 6.8 ± 0.5 training sessions per week), and had regularly participated in regional and/or national competitions in the previous year. The RR group included individuals with 3.4 ± 0.9 years of training experience, who attended 2 or 3 rowing training sessions per week (2.7 ± 0.6 h per week, 2.4 ± 0.5 training sessions per week), and reported no history of competitive sports careers. The ORS group was composed of individuals with 4.0 ± 1.7 years of training experience, who practised recreational activities different from rowing 2 or 3 times per week (2.7 ± 0.4 h per week, 2.3 ± 0.5 training sessions per week), and without history of a competitive sports career. All participants underwent clinical examinations to exclude any risk associated with maximal physical exertion during exercise. Inclusion criteria were healthy individuals reporting no disease, infirmity or underlying medical conditions (20). Exclusion criteria included having medical conditions such as diabetes, heart, respiratory or renal disease and not taking any medications at the time of recruitment. All participants and parents/guardians of minors gave written informed consent to enrol in this research. This study was conducted in accordance with the Declaration of Helsinki and approved by the CAR-IRB—University of Rome “Foro Italico” Committee (Approval No. CAR 37/2020).

### Anthropometric parameters assessment

Weight, height and sitting height were measured using a scale and a stadiometer to the nearest 0.1 kg and 0.1 cm, respectively. Body mass index (BMI) was calculated as the ratio between weight in kg and the square of height in metres (kg/m^2^). Fat mass (FM) and fat free mass (FFM) were assessed through Bioelectrical Impedance Analysis (BIA AKERN 101 Anniversary, Pontassieve, FI, Italy). Participants were measured with minimal, tight-fitting clothes and without shoes, socks or jewellery. Measurements were taken with participants in a supine position with adhesive gel electrodes at defined sites on the dorsal surfaces of the hand, wrist, ankle, and foot. Maturity offset and age at peak height velocity (HPV) was determined for each individual through the Mirwald equation which included three somatic dimensions (height, sitting height, and leg length) and their interactions (21).

### Cardiovascular assessment

Cardiovascular health was assessed through pulse wave analysis (PWA) and pulse wave velocity (PWV) using the SphygmoCor XCEL® (SphygmoCor XCEL, AtCor Medical, Naperville, IL, United States). After arriving at the laboratory, participants laid supine for five minutes in a dimly lit room before measurements of brachial systolic (BSBP) and diastolic blood pressure (BDBP) were taken. Brachial pulse pressures (BPP), mean brachial pressure (MBP), aortic pulse pressures (APP), mean aortic pressure (MAP), resting heart rate (RHR), HR period, augmentation index at 75 bpm (AIx75), ejection duration in milliseconds (EDms) and in percentage of the cardiac cycle (ED%), and subendocardial viability ratio (SEVR) were provided by the SphygmoCor XCEL software (22).

Carotid-femoral pulse velocity (cf-PWV) in m/s was assessed through applanation tonometry while the participant was laid in a supine position. The cf-PWV was calculated as the vascular distance divided by the pulse wave transit time (m/s). The vascular distance was determined by the SphygmoCor XCEL software using the distance between the carotid artery to the sternal notch, the sternal notch to the leg cuff, and the femoral artery to the leg cuff. Transit time was measured using volumetric displacement (femoral artery) and applanation tonometry (carotid artery) and distances were measured using a tape measure by a trained technician.

SphygmoCorXCEL showed a high intra-test (intraclass correlation = 0.996 and 0.983, *p *< 0.0001; cf-PWV and AIx, respectively) and a day to-day reliability (intraclass correlation = 0.979 and 0.939, *p *< 0.0001; cf-PWV and AIx, respectively). XCEL measures are valid, highly reliable and not affected by body side (23).

### Cardiorespiratory fitness assessment

Cardiorespiratory fitness was assessed through a cardiopulmonary exercise test (CPET) on an electronically braked cycle ergometer (Monark 939 E, Monark Sport & Medical, Vansbro, Sweden) and by a calibrated respiratory gas analysis system (Quark CPET Cosmed, Rome, Italy). The exercise protocol included a 1-minute resting period followed by a15 or 20 Watts per minute graded exercise test starting at 0 Watts. The participants were asked to maintain a cadence of 65–70 rpm throughout the entire test. The 15 W/min incremental exercise protocol was used for the participants in the RR and ORS group while the 20 W/min protocol for CR. HR during the exercise test was continuously recorded using GARMIN HR chest belt (GARMIN, United States ) and the rate of perceived exertion was assessed using an OMNI scale 0–10 and recorded 15 s prior to the end of every stage. The test was terminated when one of the following criteria were met: the subject achieved volitional exhaustion or a cadence of 50 rpm, a value of 10 on an OMNI scale 0–10, the 90% of the predicted HRmax (beats/min) or a respiratory exchange ratio (RER) equal to 1.1. Peak Oxygen Uptake (VO_2peak_) was calculated as the 30-second average of the highest VO_2_ during the last minute of the exercise test before the test was terminated.

### Quality control

The principal investigators completed a 3-month training period prior to the start of the project to ensure consistency with data collection and data reduction. When recording cardiovascular parameters, only PWA recordings with a quality control index equal or greater than 90% were used for the statistical analysis. Each subject received a minimum of three PWA and PWV measures with 1-minute rest in between. The results of two PWA measures reporting the lowest and closest (within ± 5 mmHg) BSBP and BDBP were averaged and used for the final analysis. The average of the two lowest and closest (within ± 0.3 m/s) cf-PWV measurements were also used (22, 24).

Prior to each CPET, a turbine calibration (using a 3-L syringe), a two-point gas calibration (16.00% and 20.93% O_2_; 5.0% and 0.04% CO_2_), a CO_2_ scrubber calibration (0.00% CO_2_), and a delay calibration were performed on the metabolic cart according to the manufacturer's recommendation. From the CPET, the raw breath-by-breath data from each test were reduced using a 3-breaths moving average (smoothing) and a 10 s time average. Then, data were imported on a shared separate Excel file for further analysis.

### Statistical analysis

All results were expressed as mean ± standard deviation. The differences in anthropometric, cardiovascular and fitness parameters among groups were tested using Univariate Analysis of Variance (ANOVA). Differences in AIx75 among the three groups were assessed by a covariance analysis (ANCOVA) model that included height as covariate. These analyses were followed by *post-hoc* analysis (Bonferroni adjustment) when significant main effects were observed. Effect size was calculated using Cohen's definition of small, medium, and large effect size (as partial *η*^2 ^= 0.01, 0.06, 0.14) (25). Statistical significance was set at *p* ≤ 0.05 and all analyses were performed using IBM SPSS statistics version 25.

## Results

Characteristics of the three groups are shown in Table 1. No significant differences between groups were observed in age, maturity offset, age at HPV, weight, BMI and body composition. However, CR showed a marginal higher height compared to RR and ORS (*p* = 0.08). CR showed higher absolute and relative VO_2_peak, and power at peak compared to RR and ORS.

**Table 1:** Characteristics of CR, RR and ORS groups (*N* = 28).

	CR	RR	ORS	*F* value	Partial eta square (*η*^2^)
Age (years)	17.6 ± 4.1	16.7 ± 2.0	15.3 ± 1.4	1.98	0.14
Maturity offset (years)	2.8 ± 2.9	1.8 ± 1.7	0.7 ± 1.1	2.75	0.18
Age at HPV (years)	14.8 ± 1.3	14.9 ± 0.8	14.5 ± 0.5	0.39	0.03
Height (cm)	183.9 ± 8.1	174.6 ± 6.7^α^	175.6 ± 8.2^α^	3.61	0.22
Weight (kg)	73.5 ± 7.7	69.9 ± 11.7	70.2 ± 18.5	0.16	0.01
BMI (kg/m^2^)	21.7 ± 1.3	22.9 ± 3.5	22.5 ± 4.5	0.23	0.02
Fat mass (%)	17.6 ± 3.1	21.0 ± 6.5	22.2 ± 6.6	1.57	0.11
Fat free mass (%)	82.4 ± 3.1	79.0 ± 6.5	77.8 ± 6.6	1.57	0.11
Nutritional Questionnaire (scores)	0.74 ± 0.07	0.75 ± 0.09	0.73 ± 0.08	0.66	0.05
VO_2_peak (ml/min)	4029 ± 827	2930 ± 322**	2714 ± 456**	14.18	0.53
VO_2/BW_peak (ml/min/kg)	54.6 ± 7.3	42.5 ± 4.2*	41.0 ± 11.0**	6.64	0.35
VO_2/FFM_peak (ml/min/kg)	66.3 ± 9.3	53.8 ± 4.5*	52.2 ± 11.4**	6.04	0.33
Power peak (watt)	267.5 ± 67.6	174.4 ± 24.0**	156.3 ± 28.2**	17.71	0.59
HRpeak (bpm/min)	187.6 ± 5.0	183.0 ± 7.3	175.6 ± 15.7	2.78	0.18
RERpeak	1.12 ± 0.06	1.11 ± 0.03	1.06 ± 0.05*	4.02	0.24

Data are presented as mean ± SD.

BMI, body mass index; CR, competitive rowers; FM, fat mass; FFM, fat free mass; HPV, height at peak velocity; HR, heart rate at exercise peak; ORS, other recreational sports; Power Peak, Power at exercise peak; RER, respiratory exchange ratio at exercise peak. RR, recreational rowers; VO_2_Peak, oxygen consumption in absolute terms at exercise peak; VO_2_/BW, oxygen consumption relative to body weight at exercise peak; VO_2_/FFM, oxygen consumption relative to fat free mass at exercise peak.

α *p *= 0.08 vs. CR.

** *p < *0.01 vs. CR.

* *p* ≤ 0.05 vs. CR.

**Figure 1:**
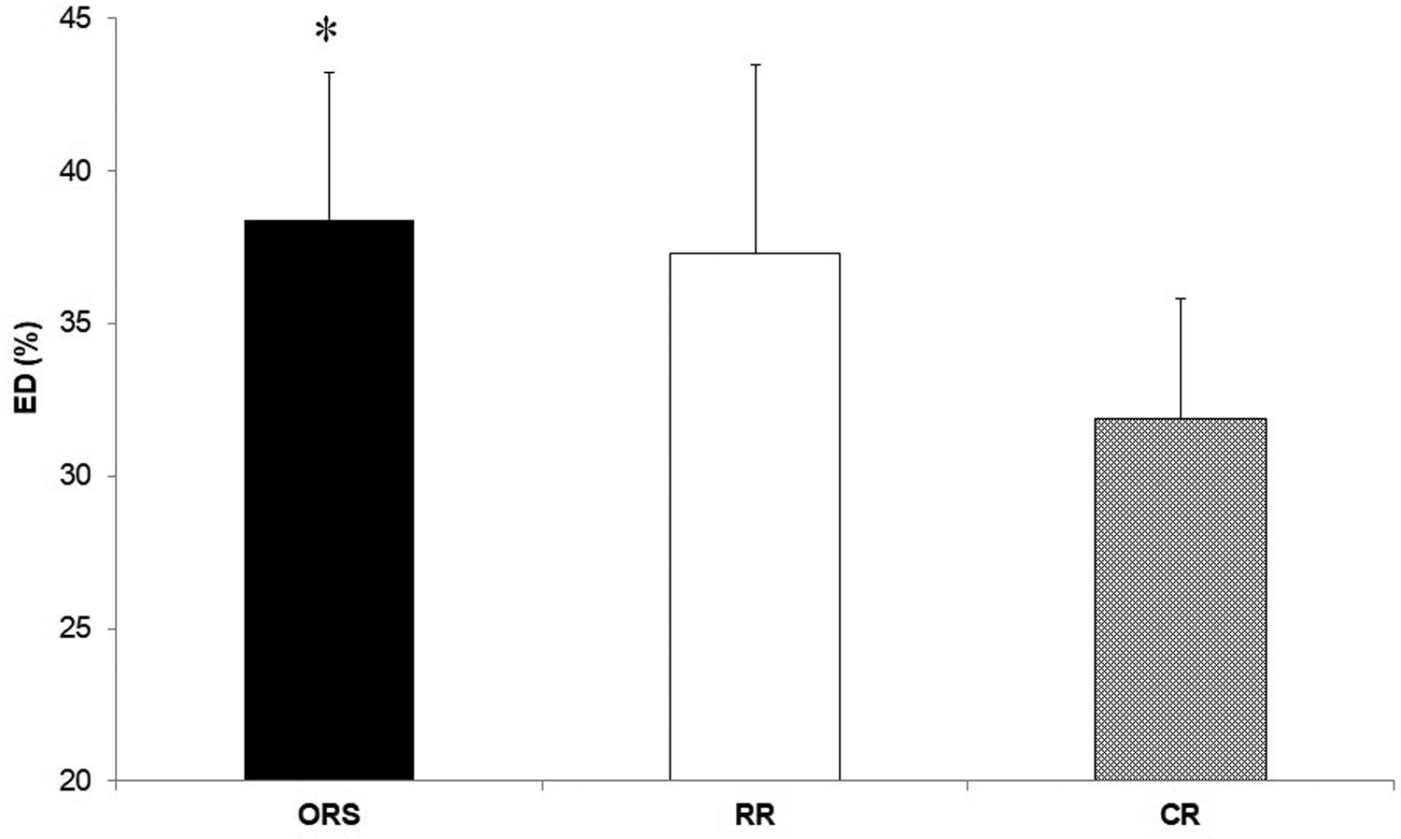
Percentage of ejection duration in ORS, RR and CR groups. *p < 0.05 vs. CR. CR, competitive rowers; ED, ejection duration; ORS, other recreational sports; RR, recreational rowers.

**Figure 2:**
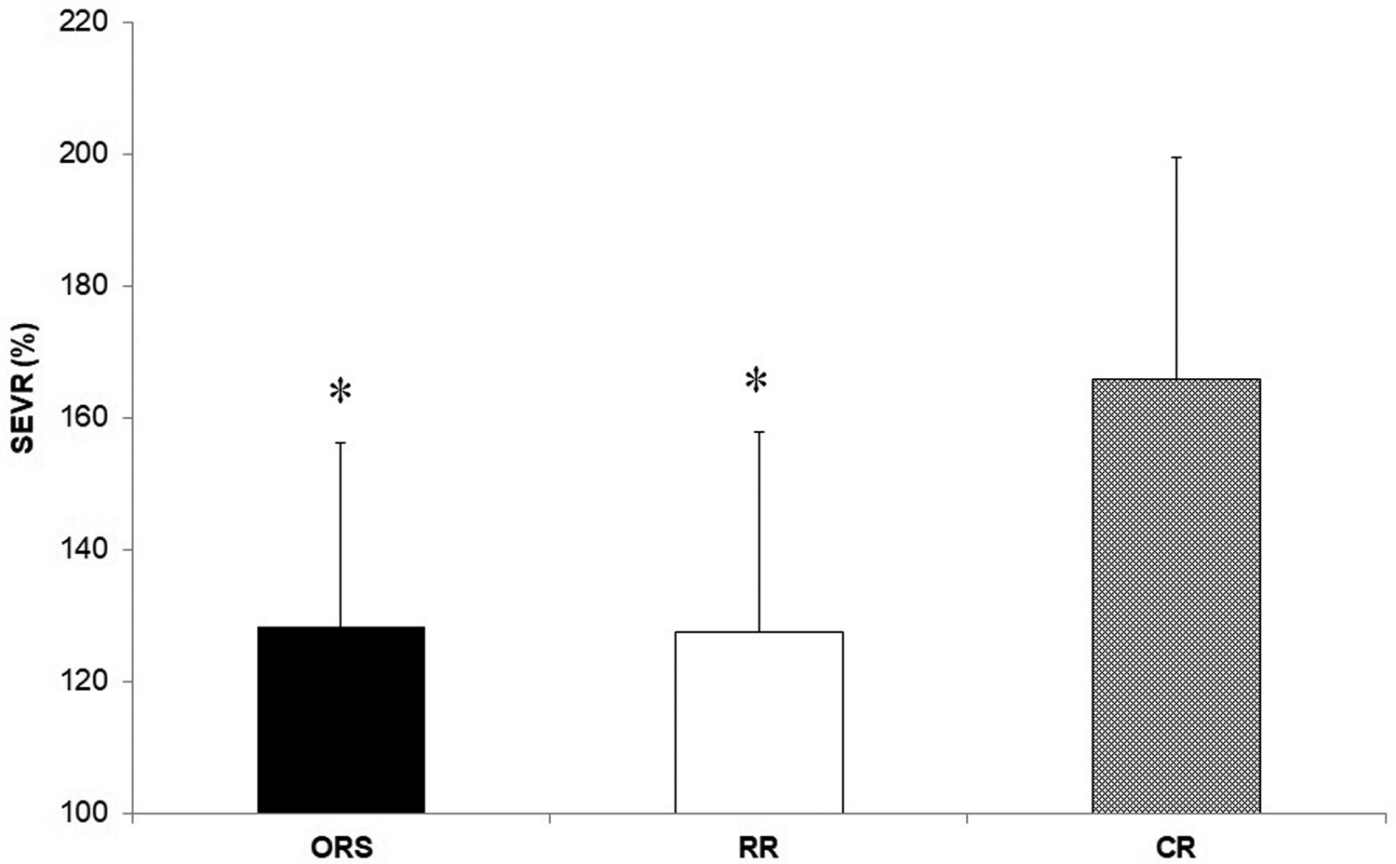
Subendocardial viability ratio values in ORS, RR and CR groups. *p ≤ 0.05 vs. CR. CR, competitive rowers; ORS, other recreational sports; RR, recreational rowers; SEVR, subendocardial viability ratio.

**Figure 3:**
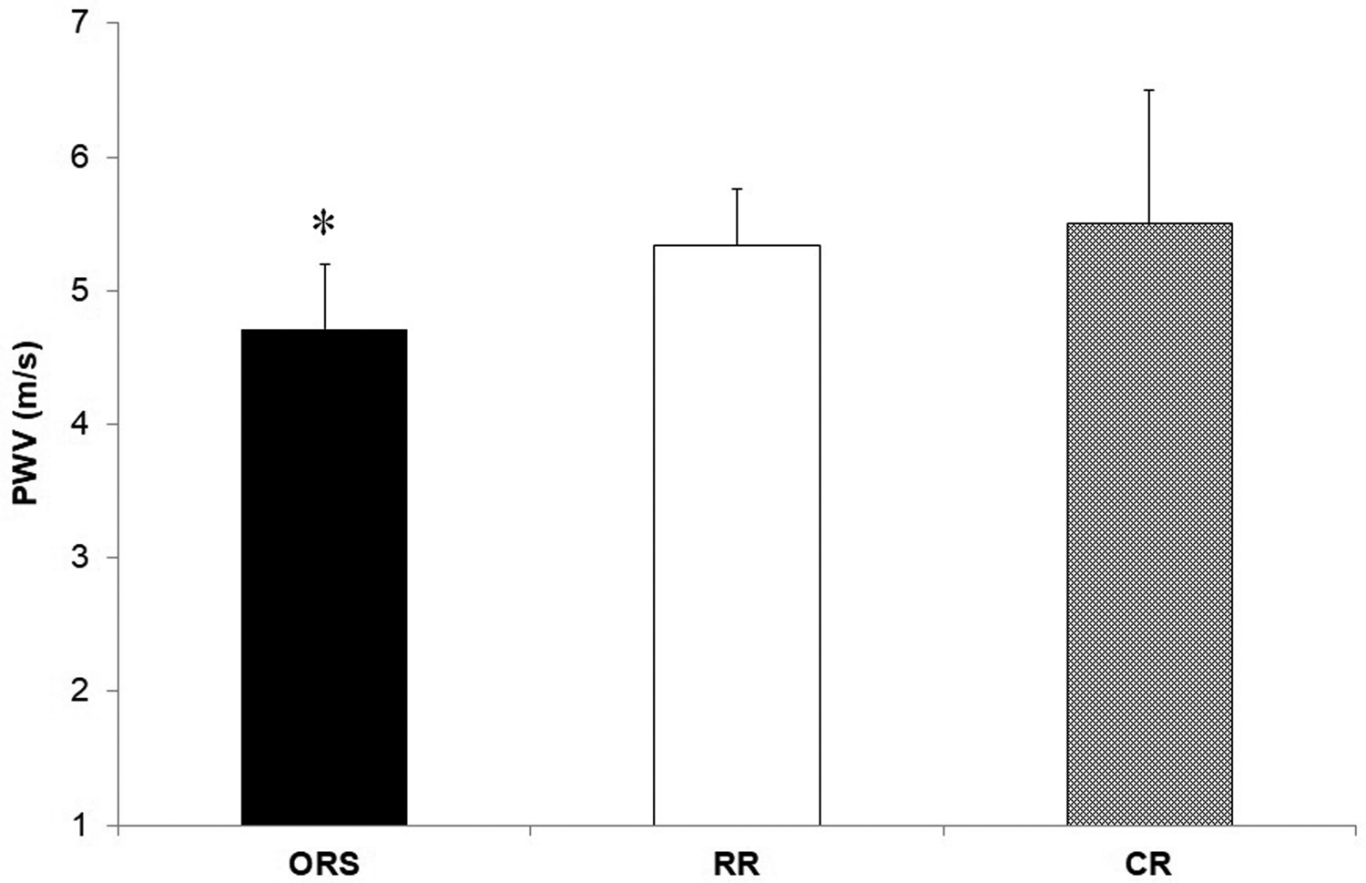
Pulse wave velocity in ORS, RR and CR groups. *p = 0.05 vs. CR. CR, competitive rowers; ORS, other recreational sports; RR, recreational rowers; PWV, pulse wave velocity.

**Figure 4:**
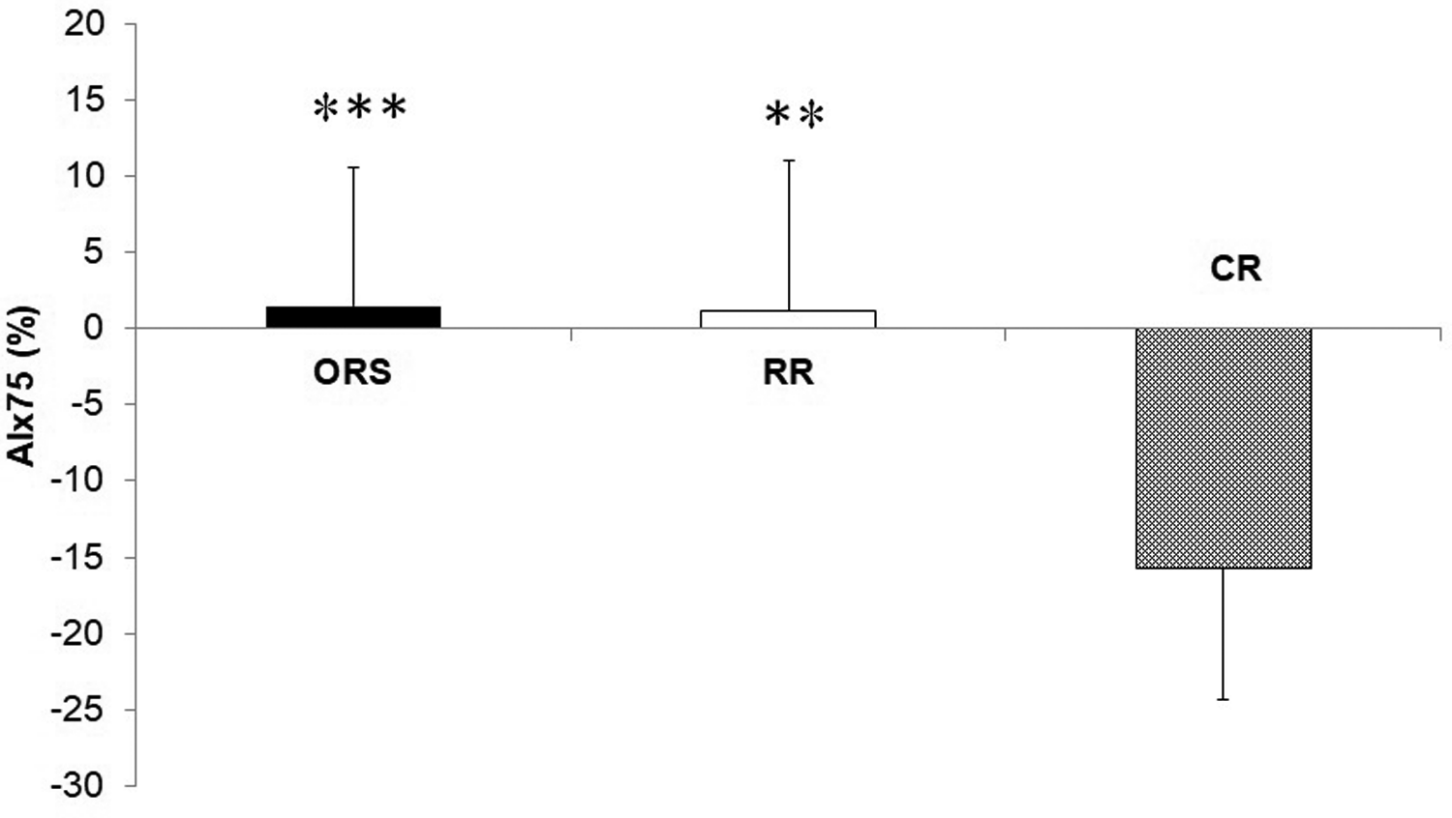
Augmentation index corrected to a heart rate of 75 bpm in ORS, RR and CR groups. ***p < 0.001 vs. CR. **p < 0.01 vs. CR. AIx, Augmentation index; CR, competitive rowers; ORS, other recreational sports; RR, recreational rowers.

Compared to ORS, CR reported lower ED% (*F*_2,25_^ ^= 4.24, *p* = 0.026, *η*^2^ = 0.25; 38.4 ± 4.8% and 31.9 ± 3.9%, respectively) (Figure 1), and higher PWV (F_2,25_ = 3.96, *p* = 0.032, *η*^2^ = 0.24; 4.7 ± 0.5 m/s and 5.5 ± 1.0 m/s, respectively) (Figure 3). Moreover, CR also reported higher SEVR compared to RR and ORS (*F*_2,25_ = 4.47, *p* = 0.022, *η*^2^ = 0.26; 165.8 ± 33.7%, 127.4 ± 30.4% and 128.3 ± 27.8%, respectively) (Figure 2) and lower AIx75 (*F*_2,24_ = 9.13, *p* = 0.001, *η*^2^ = 0.43; −15.7 ± 8.6%, 1.2 ± 9.9% and 1.5 ± 9.1, respectively) (Figure 4). No significant differences between groups were observed for brachial and aortic blood pressure, RHR, HR period and EDms (Table 2).

**Table 2:** Cardiovascular parameters in CR, RR and ORS groups.

	CR	RR	ORS	*F* value	Partial eta square (*η*^2^)
BSBP (mmHg)	117.6 ± 6.5	121.9 ± 10.2	122.1 ± 13.4	0.47	0.04
BDBP (mmHg)	60.4 ± 7.6	60.4 ± 5.0	64.1 ± 8.3	0.87	0.07
BPP (mmHg)	57.1 ± 6.3	61.4 ± 10.5	57.5 ± 9.0	0.61	0.05
MBP (mmHg)	79.5 ± 6.7	80.9 ± 5.2	83.4 ± 9.3	0.70	0.05
APP (mmHg)	36.1 ± 4.1	40.0 ± 9.3	37.9 ± 6.5	0.65	0.05
MAP (mmHg)	74.1 ± 7.1	76.4 ± 5.7	81.2 ± 8.9	2.28	0.15
HR (pbm)	57.3 ± 7.9	65.4 ± 11.6	66.3 ± 8.6	2.44	0.16
HR period (ms)	1061 ± 125	943 ± 176	920 ± 132	2.44	0.16
EDms (ms)	336 ± 25	343 ± 06	348 ± 21	0.72	0.06

Data are presented as mean ± SD.

APP, aortic pulse pressures; BDBP, brachial diastolic blood pressure; BPP, brachial pulse pressures; BSBP, brachial systolic blood pressure; CR, competitive rowers; HR, heart rate; EDms, ejection duration in ms; MAP, mean aortic pressure; MBP, mean brachial pressure; ORS, other recreational sports; RR, recreational rowers.

## Discussion

In this study, young competitive male rowers reported greater myocardial performance (ED%), higher cardiorespiratory fitness (VO_2_peak), and better indices of cardiovascular health (AIx, cf-PWV, and SEVR) when compared to recreational athletes. To our knowledge, this is the first study that investigated differences in cardiovascular functions between young recreational and competitive male rowers using non-invasive and indirect cardiovascular assessment methods.

The higher cardiorespiratory fitness in CR was expected since physiological attributes of professional rowers are among the highest recorded for any discipline (1, 2, 26). However, CR also showed a lower ED% compared to ORS (Figure 1) suggesting that continuous high-intensity exercise might result in positive myocardial adaptations in young athletes that are observable in resting conditions. Previous research showed that ejection duration is associated with high left ventricular wall thickness and cavity dimensions (13) and that greater left ventricular remodelling can occur in male adolescent rowers compared to controls (4). In addition, we found that competitive rowers showed higher SEVR compared with the two recreational groups (Figure 2) which signifies that rowing may improve the myocardial oxygen supply-demand ratio positively affecting cardiovascular health (27, 28).

Despite a large number of studies in adults, the role of training on the arteries of young rowers is still not clear (29, 30). Our results showed that PWV was higher in competitive rowers compared to recreationally trained athletes (Figure 3) and no differences in blood pressure between groups (Table 2). In clinical research, high PWV signifies higher arterial stiffness, and it is commonly associated with elevated blood pressure and RHR (31, 32). In the present study, competitive rowers showed higher PWV but lower blood pressure and HR compared to recreational athletes. This result may be explained by an improved myocardial performance, which results in a more powerful ventricular systole, rather than an increased arterial stiffness as shown by previous research conducted on highly trained individuals with enlarged heart volume and increased myocardial mass (4, 13, 33). Moreover, previous research has shown that the left ventricular ejection time can represent a potential determinant of PWV in young healthy males (34). According to these findings, the interpretation of PWV in healthy and athletic individuals should be done in conjunction with blood pressure, HR, and ED. However, more research on this topic is necessary as professional adults athletes seem not to report differences in PWV when compared to a control group (8, 16).

Another indicator of arterial stiffness that should be studied together with PWV is augmentation index (35, 36). AIx indicates the increase in aortic systolic pressure resulting from the reflected wave generated in the periphery (35, 37). In fact, competitive rowers in our study reported lower AIx75, an consequently lower arterial stiffness, compared to recreational athletes (Figure 4). These findings are in accordance with research conducted in adults which reports a significant increase in peripheral artery diameters and baroreflex sensitivity, and a decrease in vascular peripheral resistance in rowers compared to a non-athletic group (5, 7, 8). However, another study reported higher AIx in adult rowers compared to a control group underlining the need of additional investigations in this specific topic (16).

Our results also showed that the sport of rowing, when performed at the recreational level, may induce cardiovascular adaptations in adolescents that are similar to those caused by other sports. This finding is different from the results of previous research conducted on recreational adult rowers (6, 12, 14). This difference might be explained by the fact that the long-term adaptations occurring in adult rowers after years of training may not manifest in adolescent recreational athletes. However, the exercise intensity and volume used by young competitive rowers may affect cardiac functions as shown by the higher SEVR compared to their recreational counterpart even without changes in ED%. These findings are different from research in adults showing enhanced systolic and diastolic function in competitive rowers compared to their recreational counterparts (12).

In the present study some limitations need to be considered. Firstly, the relatively small sample size and the absence of a group composed by competitive athletes from other sport disciplines and of a control sedentary group. Secondly, it was possible to investigate only cardiac and vascular functioning, therefore next investigations should also assess the morphological structure of the cardiovascular system. Furthermore, future studies should use a longitudinal design to assess the course of rowers' cardiovascular adaptations from childhood to adulthood.

## Conclusions

To conclude, young competitive male rowers showed lower arterial stiffness and enhanced left ventricular performance compared to recreationally trained individuals. Moreover, PWV in healthy, competitive athletes should be interpreted differently from the one of general population and together with other indices of cardiovascular health as blood pressure, RHR and ejection duration.

## Data Availability

The raw data supporting the conclusions of this article will be made available by the authors, without undue reservation.
